# Blubber Cortisol: A Potential Tool for Assessing Stress Response in Free-Ranging Dolphins without Effects due to Sampling

**DOI:** 10.1371/journal.pone.0115257

**Published:** 2015-02-02

**Authors:** Nicholas M. Kellar, Krista N. Catelani, Michelle N. Robbins, Marisa L. Trego, Camryn D. Allen, Kerri Danil, Susan J. Chivers

**Affiliations:** 1 Protected Resources Division, Southwest Fisheries Science Center, National Marine Fisheries Service, National Oceanic and Atmospheric Administration, La Jolla, California, United States of America; 2 Ocean Associates, Inc., Arlington, Virginia, United States of America; Texas A&M University-Corpus Christi, UNITED STATES

## Abstract

When paired with dart biopsying, quantifying cortisol in blubber tissue may provide an index of relative stress levels (i.e., activation of the hypothalamus-pituitary-adrenal axis) in free-ranging cetacean populations while minimizing the effects of the act of sampling. To validate this approach, cortisol was extracted from blubber samples collected from beach-stranded and bycaught short-beaked common dolphins using a modified blubber steroid isolation technique and measured via commercially available enzyme immunoassays. The measurements exhibited appropriate quality characteristics when analyzed via a bootstraped stepwise parallelism analysis (observed/expected = 1.03, 95%CI: 99.6 – 1.08) and showed no evidence of matrix interference with increasing sample size across typical biopsy tissue masses (75–150mg; r^2^ = 0.012, p = 0.78, slope = 0.022ng_cortisol deviation_/ul_tissue extract added_). The relationships between blubber cortisol and eight potential cofactors namely, 1) fatality type (e.g., stranded or bycaught), 2) specimen condition (state of decomposition), 3) total body length, 4) sex, 5) sexual maturity state, 6) pregnancy status, 7) lactation state, and 8) adrenal mass, were assessed using a Bayesian generalized linear model averaging technique. Fatality type was the only factor correlated with blubber cortisol, and the magnitude of the effect size was substantial: beach-stranded individuals had on average 6.1-fold higher cortisol levels than those of bycaught individuals. Because of the difference in conditions surrounding these two fatality types, we interpret this relationship as evidence that blubber cortisol is indicative of stress response. We found no evidence of seasonal variation or a relationship between cortisol and the remaining cofactors.

## Introduction

Glucocorticoids (GC), such as cortisol, are one of the most commonly used indicators of stress-response activation in wildlife and are increasingly being used to assess overall well-being of populations [1,2]. These steroid hormones are produced from the activation of the hypothalamic-pituitary-adrenal (HPA) axis in response to perceived stressors and enable an animal to counteract or evade dangerous circumstances [2,3]. Typically, this response lasts from minutes to hours [3,4]; however, prolonged or frequent activation of this pathway can lead to detrimental effects on survival and reproduction [5–7]. As such, frequent observations of abnormally high GC levels within populations are generally interpreted as indicative of compromised health [6,8–10], although it should be noted that both abnormally high and low levels are associated with compromised health. It is therefore important to obtain reference levels from healthy populations.

Though GC levels typically have been measured in blood samples, the use of other sample matrices is expanding rapidly [1,11]. Sample accessibility is one of the chief reasons for cetacean researchers to choose samples other than blood. Blood collection is limited to very unique situations in which individuals can be captured (i.e., shallow water embayments and near shore habitats); these capture operations are logistically challenging and very expensive for collections of relatively few samples. An alternative is skin sampling; skin biopsies are one of the most common biological samples collected from free-ranging cetaceans [12–14]. These samples have traditionally provided information about diet (lipid composition, stable isotopes), pollutant accumulation, and genetics [14]. Increasingly, these samples are also being used to study the physiological states of sampled individuals by measuring lipophilic hormones within the fatty blubber tissue connected to most biopsies. Recent studies have shown that reproductive steroid hormones, chemically similar to cortisol, can be measured from the blubber obtained from remote biopsies [15,16]. For example, blubber progesterone level has been a robust indicator of pregnancy due to the 20–60 fold mean difference in concentration between pregnant and non-pregnant females [17–19], a finding that is consistent across a wide range of cetacean taxa [20]. Likewise, blubber testosterone has been used as an indicator of male cetacean reproductive activity [21].

In addition to sampling accessibility, there may be added advantages for measuring stress hormones from blubber tissue. Studies evaluating GC levels, especially those using blood samples, can be constrained because the act of capture and sampling (i.e., blood draw), can elicit an elevation in GC concentrations, making it difficult to discern whether the stressor of interest or the sampling event is primarily responsible [22–25]. In the literature, the evidence is mixed on whether capture events raise blood cortisol concentrations in wild dolphins, especially in the first minutes after capture [26–28]. Blood samples taken within 10mins of stress response initiation are exceedingly rare, and it is unknown whether the events before the start of capture (e.g., boats following target individuals) also trigger a response. Adipose tissue, like that found in blubber, amasses steroid hormones in high concentrations as the hormones passively diffuse from capillaries into this mostly lipid environment [29–31], where processes that would break down or remove them are slow relative to those occurring in the blood. Therefore, the dynamics of blubber hormone composition are much less rapid than changes in blood. Consequently hormone signals in blubber represent longer windows of physiological time and are not heavily influenced by the events that take place in the minutes immediately prior to sampling. However, the timing of this lag is not yet known.

In this study, we measured cortisol concentrations in blubber samples from short-beaked common dolphins, *Delphinus delphis*, incidentally killed in the California drift gillnet fishery or stranded dead on the beach within San Diego county. The fishery is active within the core of the regional *D*. *delphis* stock from late summer/ early fall until the end of January [32]. This fishery’s estimated annual mortality for *D*. *delphis* is on average ~120 individuals/year [32]. Approximately 3.5 reported *D*. *delphis* strand dead each year along the San Diego coastline, with disproportionately more animals stranding during the spring and summer [33]. Though the *D*. *delphis* stock within the study area appears to be stable in abundance, threats in the region include predation, incidental bycatch, harmful algal blooms, prey loss, contaminants, acoustic perturbations, and generalized environmental disturbance [33–38].

The intent of this investigation was to develop and validate a method to accurately measure blubber cortisol concentration in *D*. *delphis* and to assess its variation with respect to life-history state, general cause of mortality (stranded or bycaught), season, and adrenal mass. Blubber cortisol was measured in 63 animals representing specimens collected from 1) strandings along the San Diego county coastline and 2) incidental gillnet bycatch in the Southern California Bight. The blubber cortisol measurements were validated using standard assay quality assurance tests including parallelism analysis, matrix interference assessments, and a general biological validation.

## Materials and Methods

### Ethics Statement

All samples for this study were collected postmortem after carcasses were recovered by the National Marine Fisheries Service (NMFS) Southwest Region Marine Mammal Stranding Network or the eastern tropical Pacific purse-seine and California/Oregon Gill net Observer Programs. No animals were directly targeted and killed for this or any other associated study. Specimens collected by both of the observer programs were incidentally killed in fishery nets. Incidental bycatch of non-threatened marine mammals is permitted through the NMFS Marine Mammal Authorization Program under the Marine Mammal Protection Act (16 U.S.C. 1371(a)(5)). Response to and sample collection from dead, stranded animals by NMFS is covered under the MMPA (16 U.S.C. 1421).

### Samples

Two sources of blubber tissue were used in this study. The first was composed of dead-stranded animals (n = 23) collected from the San Diego county coastline by the Southwest Fisheries Science Center Stranding Program (1996–2013). Each stranded individual was given a “stranding code” designation to represent the decomposition stage, where code 1 = live stranding, 2 = fresh stranding, 3 = moderate decomposition, 4 = advanced decomposition, 5 = mummified carcass (no specimens with a code 5 were used in this study). The second group of blubber tissue samples were taken from dolphins incidentally killed in the California drift gillnet fishery (n = 40) and collected by observers in the California/Oregon Gillnet Observer Program, between 1991 and 2011. Specimen total length, sex, adrenal mass, maturity status, pregnancy state, and lactation status were recorded; for stranded animals, specimen condition (“carcass classification” as per Geraci and Lounsbury [39]) was also noted. Animals were considered sexually mature when males had right testes sizes greater than 200g [21] and females had corpora counts greater than one [39]. Pregnancy state and lactation status were determined by the presence of conceptus/fetus and milk, respectively [39]. Sexually mature females that were neither classified as lactating nor pregnant were designated as “resting” for this manuscript. All blubber samples were taken from the dorsal, mid-thoracic area on the left side of each animal. In each case, the epidermis (including dermal papillae) was removed and all the remaining tissue, from the proximal end of the dermal papillae inward through the main dermis and the hypodermis, was retained for hormone processing (all this tissue is referred to as “blubber” for this article). It should be noted that none of the stranded animals showed evidence of human interactions, including signs of fishery entanglement, prior to death.

### Blubber cortisol extraction

The blubber hormone extractions followed the methods delineated in Kellar et al. (2006) with several modifications to simplify the procedure and increase consistency. Approximately 0.075 g—0.15 g of blubber were homogenized six times at a speed of 5 m/s for 45-second intervals. The contents of the homogenization tube were poured into a glass tube (T1), the homogenization tube was then rinsed with 500 μL of ethanol, and the washed contents were transferred into T1. The homogenate:ethanol solution was then separated from the grinding media and placed into a new glass tube (T2). The reminding contents of T1 (with grinding media) were rinsed again with another 500 μL of ethanol and combined with the homogenate in T2, thenthis step was repeated once more. The homogenate/rinse solution combination was combined with 2 mL of 4:1 ethanol:acetone. The resulting solution was vortexed and then centrifuged at 5000 rpm for 15 min. The supernatant was transferred and evaporated. Two milliliters of diethyl ether were added to the evaporated contents, vortexed, and centrifuged again. The supernatant was collected and evaporated, and the residue was resuspended in 1500 μL of acetonitrile, vortexed, and 1500 μL of hexane added to the mixture. After the solution was vortexed and centrifuged again, the acetonitrile layer was aspirated into a new tube and the process was repeated with another 1500 μL of hexane. The final portion of acetonitrile was collected and evaporated. The remaining residue was centrifuged at 5000 rpm for five minutes and stored at -20°C.

### Cortisol enzyme immunoassay

To prepare the samples for the enzyme immunoassay (EIA), they were suspended in 250 μL of 1M phosphate buffered saline and then vortexed in the multitube vortex for 15 min. We used an EIA kit K003-H1 (Arbor Assays, Ann Arbor, MI, USA) that has 100% reactivity with cortisol, 18.8% reactivity with dexamethasone, 7.8% reactivity with prednisolone (1-Dehydrocortisol), and 1.2% reactivity with both corticosterone and cortisone. The total assay range was between 100 and 3200 pg/mL with the 20%–80% B/Bo range typically between 200 and 2000 pg/mL. Therefore, samples that exceeded this range had to be diluted further to be accurately measured. These samples were diluted further depending on their original EIA measurements such that the final measurements would fall within the range of the control samples. The intra-assay coefficient of variation (CV) was between 6.0% and 14.7%, and the inter-assay CV was between 7.2% and 10.9%.

We determined the extraction efficiency using spiked samples as described in Kellar et al. (2006). The extraction control samples were spiked with 200ng of cortisol. The extraction efficiency was calculated as the amount of quantified cortisol (via enzyme immunoassay analysis) of the spiked samples minus the quantified amount in the non-spiked samples, all divided by the original amount of cortisol added (spiked) before the extraction. The percentage of cortisol that was recovered after extraction was calculated and each assay value was adjusted to the standard prior to analysis. The variation of the extraction efficiency was added to the inherent sample measurement variation such that the reported values represented greater variation for each statistical stratum than was true in the measurements alone.

### Parallelism and matrix effects analyses

We conducted two additional quality control assessments to gauge the performance of using the blubber extracts with the cortisol EIA kit. The first was a parallelism test in which a serially-diluted pool of sample extracts was run along with the standard controls of the assay to determine whether the linear decrease in measured values of the pooled sample was parallel to the standard curve, an indication that the assay is measuring the same antigens in the blubber as in the standards. Extracts from four individuals (3 female, 1 male) were pooled together to obtain a representative sample across our sampled individuals. The pooled sample concentrations were made by diluting four times from the neat preparation to 1/16 decreasing by a factor of two. Each dilution was run three times, and the resulting curve of the detection metric (optical density of the sample/optical density when no sample is added (B/Bo)) as a function of the dilution state was then compared to the standard curve using a bootstrapped stepwise linear slope analysis.

The second quality assessment examined the potential matrix interference, the effect of the blubber extract itself on the measurement of cortisol. A standard solution (final concentration 200pg/μL) was spiked with either phosphate-buffered saline or a set of serial dilutions of a pooled sample (4–160μL of the pooled extract was added to the 200pg/ml solution, n = 9) composed of four blubber cortisol extracts (from four different individuals) to make a final equivalent volume of 240μL (with 48pg of cortisol added from the standard solution). Each of the nine serial dilutions was run in duplicate. The concentration of cortisol contributed from the pooled sample (neat = ± 56.5pg/ml) was subtracted from each sample-spiked measurement so its contribution would be factored out of the assessment. A simple linear regression was used to determine if there was a significant relationship between deviations from expected concentration and increasing level of spiked blubber extract concentration. MATLAB R2009b (Mathworks, Natick, MA) was used to perform all above data analyses.

### Blubber cortisol cofactor analysis

We assessed eight factors as potential correlates with blubber cortisol concentration. These were: 1) fatality type (i.e., stranding or bycatch), 2) specimen condition (stranding code 1–4), 3) total body length in cm, 4) sex, 5) sexual maturity (i.e., immature or mature), 6) pregnancy state (i.e., pregnant or non-pregnant, 7) lactation status (i.e., lactating or not lactating), and 8) adrenal mass in g. Total length is in essence used as a proxy for age; though sexual maturity also has an important age component, it was determined by the authors as a potentially endocrinologically important distinction that might be otherwise lost within a distribution of ages or lengths. Season was also examined, but because of its cyclical nature, we conducted its analysis separately with a different methodology (see below). Each of these factors’ predictive ability was assessed as part of a Bayesian model averaging procedure (with a generalized linear model framework) using a method in which the model estimation process also selects which factors to include and which to exclude for each iteration of a Markoff chain Monte Carlo (MCMC) run [40]. In this analysis, sets of logistic generalized linear models were constructed in WinBUGS [41] with each of the eight factors multiplied by a factor-specific Bernoulli selection parameter in the form:

$$c_{i}\sim a+\sum y_{j}\times b_{j}\left(x_{ij}\right)$$

where *c*_*j*_ was the blubber cortisol concentration for individual *i*, a was the model intercept, *b*_*j*_ was the coefficient of factor *j* and *γ*_*j*_ was the selection parameter for each factor with a probability of *p(j)*, equal to the mean of *γ*_*j*_. A log link function was used, as blubber cortisol measurements were qualitatively assessed to be log normal. Vague priors were set for all marginal slope coefficients from normal distributions, each with mean = 0 and variance = 1,000 (note: prior distributions where variance >1,000 did not converge). The prior on each selection parameter was set to be p = 0.5. The weight (an indication of importance) that each factor had on the estimate of the sampled animal’s blubber cortisol was equal to *p(j)* and directly proportional to the number of iterations that particular factor was selected in the MCMC chain. Marginal posterior probabilities of the selection parameters with median values greater than p = 0.5 were those in which the weight of evidence supported their inclusion in the model. The data for each of these cofactors were normalized (mean = 0 and a standard deviation = 1) prior to analysis.

### Blubber cortisol seasonality analysis

We employed a multiple step process to assess the seasonal variation in blubber cortisol in efforts to create a robust data set with an appropriate temporal scale. Average monthly values of blubber cortisol were estimated from the stranded carcasses alone because they were collected year-round versus bycatch samples which were only collected during the active commercial gillnetting season (typically September to January). Seasonality of the blubber cortisol was assessed using a permutation test of the mean concentration for a moving-three-month average in which any one month’s mean concentration was estimated from the three moving-window means in which that month was contained. For example, March was calculated as the average of each of these three periods: 1) January-February-March, 2) February-March-April, and 3) March-April-May periods. This process minimized unrealistic drastic month to month variation without grouping by arbitrary static three-month periods. It resulted in a vector of 12 estimated monthly-mean-blubber-cortisol values. Using this same running-mean (or moving-window) process with our sample set randomized 10,000 times with respect to month (keeping the number of observations the same for each month), we generated a 12 x 10,000 matrix representing the expected null distribution for each month [42]. The observed mean value for each month was then compared to each month-specific null distribution to detect any signification deviation of blubber cortisol (observed values outside the 95% probability envelope of the null distribution) with respect to month.

## Results

**Fig 1:**
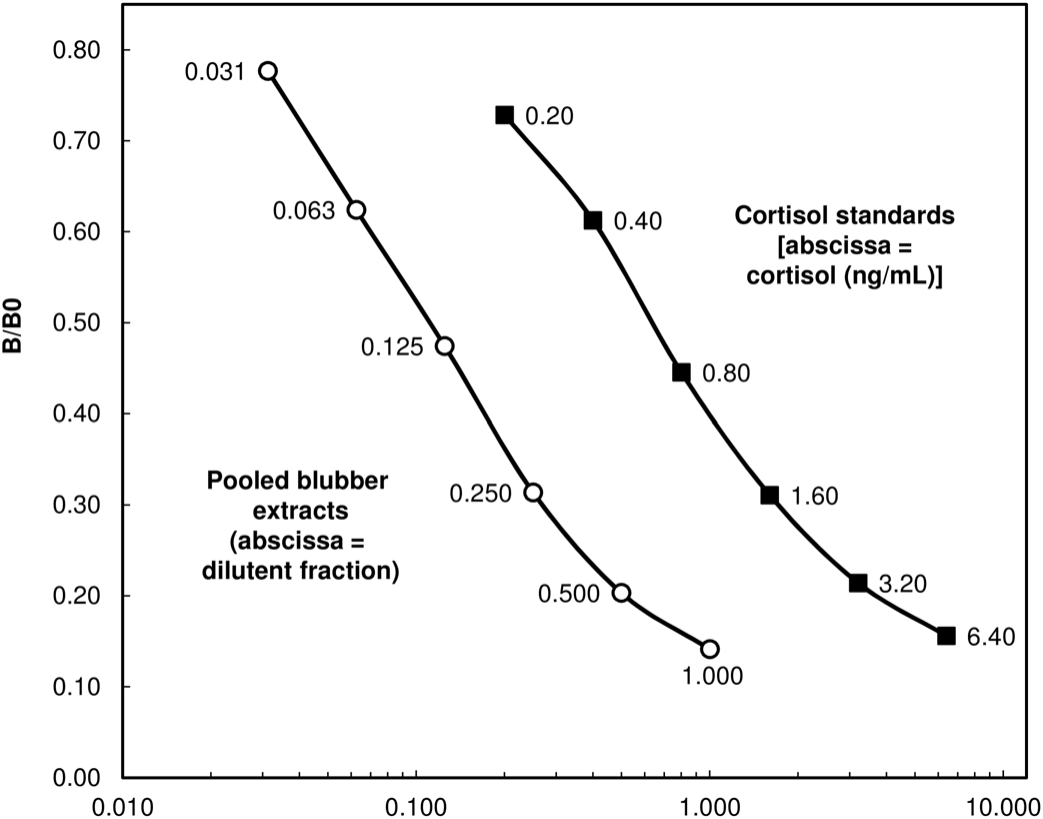
Results from linearity assessment of cortisol enzyme immunoassay (EIA) with blubber tissue extracts. Serial dilutions of extracts (open circles) show parallelism with the standards of the cortisol EIA (dark squares) (observed/expected = 1.01, 95%CI: 99.3–1.06); an indication that the assay is measuring the same antigens in the blubber as in the standards and therefore suitable for use with the short-beaked common dolphin blubber tissue extracts. Four individuals were represented in the pooled blubber extracts; 1 male and 3 female.

**Fig 2:**
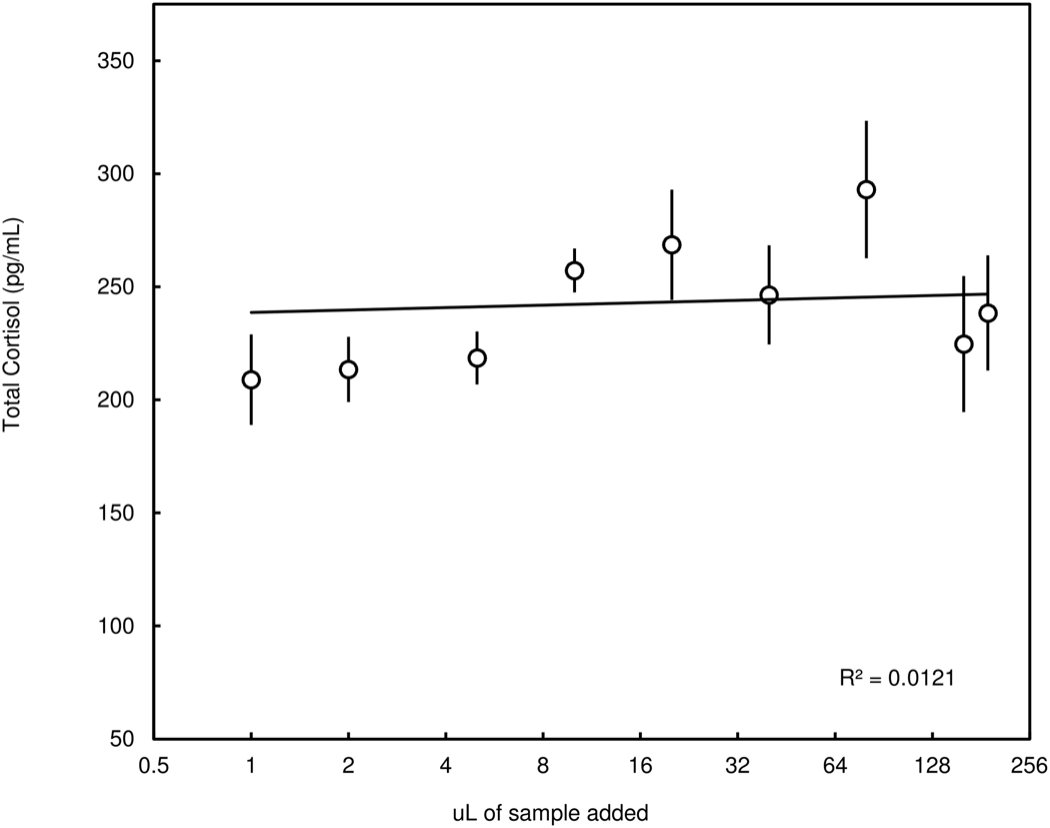
Results from matrix interference assessment. A standard solution (final concentration 240pg/ml) was spiked with either phosphate-buffered saline or a set of serial dilutions of a pooled sample (“uL of sample added”) composed of blubber cortisol extracts from four individuals to make a final equivalent volume of 240ul. The concentration of cortisol contributed from the pooled sample (neat = ± 398.5pg/ml) was subtracted from each sample-spiked measurement so its contribution would be factored out of the assessment. Little to no evidence of matrix interference was observed (r^2^ = 0.012, p = 0.78).

The estimated extraction efficiency (68.5% +/- 13.9% SD) was based on 20 measurements of recovery of 200 ng/ml of cortisol (Fluka Hydrocortisone Analytical Standard 31719; Sigma-Aldrich, Munch, Germany) spiked blubber extracts. This measured extraction efficiency was in turn used as a correction factor applied to all blubber cortisol measurements within this study. The sensitivity was estimated as the absorbance (mean OD + 2 SD) of the zero standards (B_0_, 5 runs), equating to approximately 22.1 pg of cortisol per g of blubber in 100mg blubber sample. The EIA standards and the pooled serially diluted blubber extracts exhibited statistical parallelism (Fig. 1, r^2^ = 0.941, slope = 1.038) when analyzed via a bootstrapped stepwise parallelism analysis (observed/expected = 1.03, 95%CI: 99.6–1.08). We found no significant trend in expected concentration when our standard solution was spiked with increasing amounts of blubber extract (Fig. 2, r^2^ = 0.012, p = 0.78); a finding consistent with little or no evidence of matrix interference. Finally, no significant relationship was found between storage time of blubber samples at—20°C and measured cortisol concentration in *D*. *delphis* blubber samples (r^2^ = 0.0243, p = 0.610).

**Fig 3:**
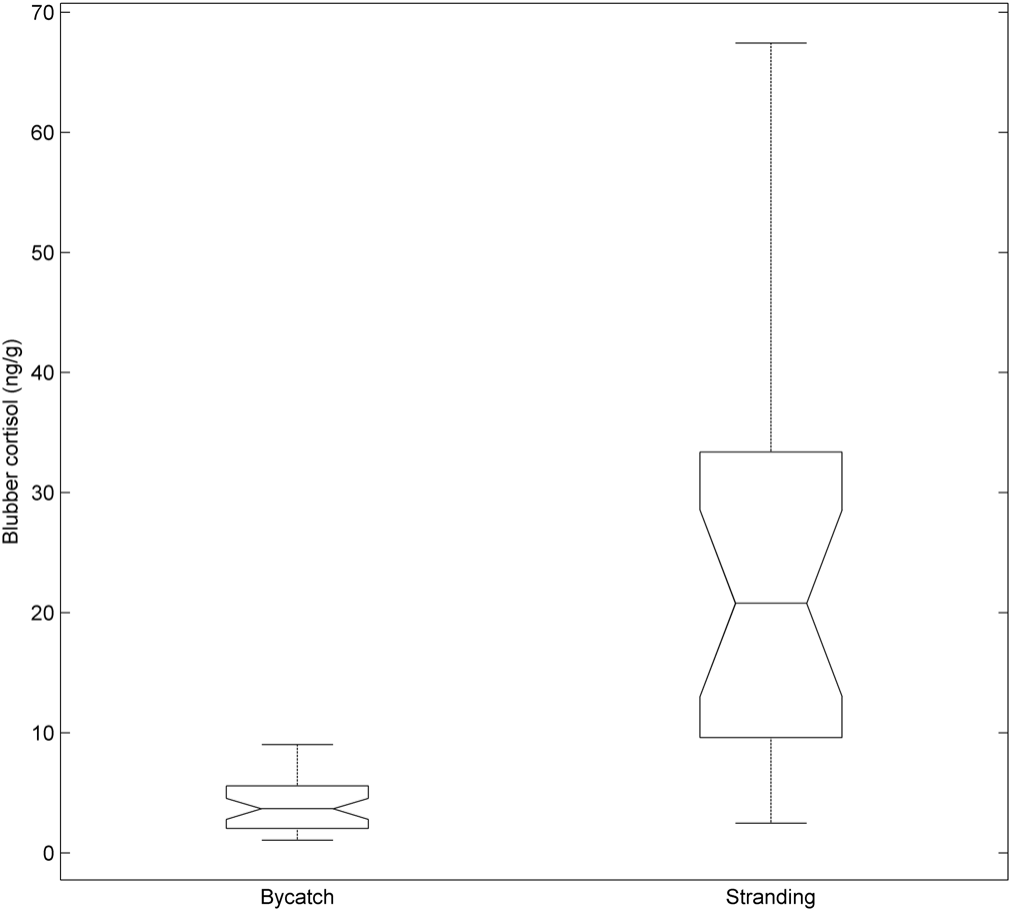
Blubber cortisol concentrations for bycaught (n = 40) and beach stranded (n = 23) *D*. *delphis*. Horizontal box lines represent the lower quartile, median, and upper quartile values. Whiskers lines indicate range of concentrations. Points of inflection represent upper and lower bounds to the 95% confidence interval. Stranded individuals (24.3 ng/g) had on average 6.1 times more blubber cortisol than bycaught animals (3.99 ng/g).

The cofactor analysis revealed that fatality type was the only factor that varied with respect to blubber cortisol concentration. Note for this analysis if a factor’s inclusion rate is above 50%, the weight of evidence indicates that models with that factor are more predictive of blubber cortisol level than those without that factor: 1.0% = strong evidence that a factor is not predictive, 99.9% = strong evidence that factor is predictive. We found a large disparity in the inclusion rate of the different factors, with fatality type selected in 99.99% of the iterations; the rest of the factors were selected for inclusion less than 1.0% of the time (Table 1). This was primarily driven by the finding that stranded animals (24.3ng/g) had on average 6.1 (Fig. 3, S1 Data) fold more blubber cortisol compared to bycaught ones (3.99ng/g). The variation of these cortisol measurements from the stranded animals was also much larger than that found among bycaught animals, even after log transforming the measurements to reduce heteroscedastic variation.

**Table 1:** Model averaged coefficients for factors associated with blubber cortisol, median, and 95% probability interval values.

Factor	Model coefficients			
	2.50%	median	97.50%	%selected
Bycatch / Stranding	0.559	0.731	0.903	99.99%
Sex	-0.266	0.093	0.08	0.51%
Maturity	-0.241	-0.058	0.119	0.33%
Length	-0.235	-0.061	0.129	0.01%
Adrenal mass	-0.263	-0.033	0.202	0.40%
Condition	-0.281	-0.051	0.175	0.41%
Pregnancy	-0.188	-0.001	0.19	0.31%
Lactation	-0.065	0.124	0.312	0.68%

% selected: percent of the iterations that each factor was selected to be included in the final model. Those with positive median values indicate direct relationships and negative values indicate inverse relationships. The evidence is indicative of a strong relationship between blubber cortisol and the factor “fatality type” (Bycatch/Stranding) and it was selected for inclusion in more than 99.99% of the model averaging iterations. There was no evidence (< 1.0%) for inclusion for the rest of the factors.

Fatality type (e.g., stranded or bycaught) was such an overwhelming correlate of blubber cortisol level that we also ran the model averaging procedure while limiting the analysis to solely bycaught individuals. This eliminated fatality type as a factor (also “stranding code or condition”) and reduced the effects of the variation in blubber cortisol associated with stranding. The results show none of the remaining six factors were included more than 1% of the time, indicating that there was no discernable evidence that blubber cortisol varied by sex, sexual maturity state, pregnancy status, lactation state, total length (a rough approximation for age), or adrenal mass. This finding is corroborated by the relative similarity of concentrations among all the demographic groups in the bycatch dataset, with all demographic group mean values estimated between 2.4 and 5.2 ng/g (Table 2).

**Table 2:** Mean cortisol concentrations in the blubber of immature and mature male and female ***Delphinus delphis*** (bycatch specimens) of various reproductive states.

Class	Female			Male		
	Mean ± SE	Range	N	Mean ± SE	Range	N
Immature	3.7 ± 2.3	1.1–7.0	6	4.3 ± 1.4	2.1–6.7	8
All mature	4.2 ± 2.2	1.2–8.2	16	3.7 ± 2.9	1.3–9.0	10
Resting	2.4 ± 1.2	1.4–3.8	5			
Lactating	5.2 ± 1.8	2.3–7.7	6			
Pregnant	4.7 ± 2.3	1.2–8.2	6			
All classes	4.0 ± 2.2	1.1–8.2	22	3.9 ± 2.3	1.3–9.0	18

The concentrations are corrected for extraction efficiency (see text) and are reported as ng of cortisol per g of blubber extracted.

**Fig 4:**
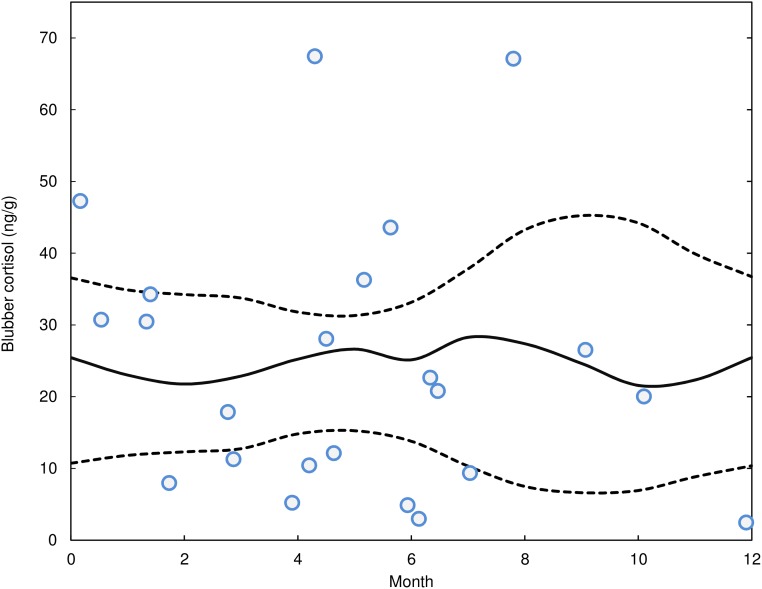
Results of a seasonal analysis of blubber cortisol levels in stranded *D*. *delphis*. The open circles represent cortisol concentrations for each individual. The observed three-month-running averages were calculated for each month (see text) and represented by the solid line. The dotted lines represent the 95% confidence envelope for the total seasonal randomness, i.e., the cortisol measurements were randomized relative to sampling date in 10,000 permuted datasets from which three-month-running averages were calculated. All observed three-month-running average values (represented by the solid line) are contained within the 95% confidence interval of the randomized null distributions indicating there was no evidence of seasonal fluctuations in blubber cortisol value.

There was also no evidence that blubber cortisol concentrations varied by season in the stranded animal data set. The running monthly average concentrations all fell well within the 95% confidence envelope and there is no evident trend in these values (Fig. 4). Moreover, there was very little difference in the observed averages, with lowest estimated mean values found in February (21.8ng/g) and October (21.6ng/g) and the highest values in August (27.4ng/g) and May (26.6ng/g).

## Discussion

The evidence of this study suggests the mode in which an animal dies is by far the primary driver of its post-mortem blubber cortisol levels. Increased activation of the HPA axis during the events that lead to an animal’s death is to be expected in most instances, especially in those in which an animal is cognizant of the threat to its life [43,44]. We believe the timing of the accumulation of cortisol in the blubber relative to these different fatality types is what is driving the differences in observed blubber cortisol levels. Because blubber is more peripheral to the production of cortisol than blood, which is directly receiving the hormone as it is produced from the adrenals [45], the expectation is that peak cortisol levels in the blubber will lag those in the blood and there is evidence that this is true for other steroid hormones found in the cetacean blubber [17], though an estimation of the duration of this lag has not yet been published. Moreover, the volume of cetacean blubber is higher than that of the blood [46–49], and steroids appear to accumulate in higher concentrations per unit volume in the lipid-laden blubber tissue compared with those in the aqueous of blood [50,51]. Together these three conditions of the blubber (peripheral tissue, large volume, and high cortisol accumulation rate) indicate that levels in the blubber are likely a function of both total cortisol production and the duration of production; in essence an integration of cortisol production over a particular time period. This is similar to what is found for other tissue matrices like hair or feathers in terrestrial vertebrates [1].

We posit that the time from perceived threat to death is on average much greater in dead stranded animals than those that die as bycatch, and that ultimately leads to the large differences we see between fatality types. Moreover, it is to be expected that *within* each fatality type that there is likewise substantial relative variation in this length of time. If this variation is largely independent of demographic state, then it is plausible that any inherent differences in blubber cortisol level associated with different demographic states (and other factors such as adrenal mass and season) are masked by those caused by the variation in the time of perceived threat before death. This could also be true for animals that die as bycatch; however the variation in that time is much more limited by specific circumstances associated with fishing activities. The logical presumption is that drowning (or suffocation) is the dominant cause of death for dolphins fatally ensnared in gillnets [52,53] (note there is ongoing debate about whether bycaught cetaceans either drown or suffocate (i.e., die without inhaling water into the lungs)[52]. It is also logical to assume that these animals are disproportionately otherwise healthy at the time of death compared to animals obtained via beach stranding. Depending on when during a dive an animal becomes ensnared in a fishing net (and the ability of that an animal to obtain air while ensnared, if any), the time from perceived threat to death would be on the order of tens of seconds to tens of minutes. For animals that ultimately strand on the beach (alive or dead at initial stranding), the time of perceived threat until death likely varies much more. This duration ranges from almost instantaneous deaths for which there is no threat perception (some ship strikes and predator attacks) to long term illnesses and poor health conditions in which an animal can be cognizant of threat to survival for many days, This range includes gillnet fishery entanglement, though again the stranded specimens we used showed no physical evidence of entanglement prior to stranding. Overall, it is reasonable to assume that for these stranding deaths, perception-of-threat duration on average is much longer, and this time varies more greatly than for bycatch fatalities. The data in this study supports this premise, showing much higher mean blubber cortisol levels in stranded animals and much greater variation than what we see within the bycaught individuals.

An alternative potential explanation is that the cause of death, instead of the duration of impending death, is driving the differences seen between bycaught and stranded blubber cortisol levels. Because strandings are disproportionally composed of starvation and illness fatalities, the physiology associated with nutritional deficits may instead be the primary driver of the large differences in blubber cortisol levels between strandings and bycatch. One example can be highlighted in cortisol’s important role in energy regulation and its stimulation during periods of substantial nutritional deficits [54,55]. Animals experiencing starvation fatalities likely have prolonged cortisol activation that would be more driven by extreme energy regulation than perceived threats to self. Moreover, it is reasonable to assume that many deaths due to illness lead to foraging performance declines such that, though the ultimate cause of death is not starvation, there is a sufficient nutritional deficit before death to cause prolonged elevated cortisol levels in the blubber. Of course, both nutritional deficits and duration of perceived threat may be contributing to blubber cortisol levels. Currently, they are confounding factors that we are unable to resolve with this dataset.

In the past, dead specimens have been used for biological validation of steroid hormones as markers for specific conditions (pregnancy, maturity state, etc.) [17–21]. They are extremely useful because of the rich auxiliary data regarding age, reproductive state, condition, size, and so forth. However, because the levels of GCs in dying animals can often change radically due to the conditions associated with the cause of death, it is more challenging to use measurements from these animals to characterize baseline cortisol levels for free-swimming healthy individuals as we have done for other non-stress related steroid hormones. Therefore we do not recommended interpreting these as baseline levels, even those from the bycaught animals, until a better characterization of the dynamics of cortisol accumulation in the blubber are resolved and we have a better idea of the timing and duration of death events. Dart biopsies may avoid these issues, but much less auxiliary information is obtained at collection, and there is evidence that the dart biopsying itself may alter lipid composition in the samples acquired using this method [56].

Many studies across vertebrate taxa have found that baseline and stress-induced GC levels vary with respect to sex, age, reproductive state, and season in various sample matrices [9,57–61]. Typically GC levels increase with age, and females, especially pregnant and lactating individuals, have higher levels than males [9,59,62,63], though there are significant interspecies differences in this regard. Most species show a peak in baseline glucorticoid secretion during the breeding season [59,64,65], though again species-specific differences are common. We found no evidence of dolphin blubber cortisol variation in relation to these differing demographic subsets. We suspect that the effects of unaccounted variation associated with the specific circumstances or conditions of each fatality (e.g., variation in time from perception of threat to death; even within the bycatch animals, there was likely variation on the order of 10’s of seconds to 10’s of minutes from perceived threat to death) masked any variation of inherent differences specific to demographic status or season. Another likely explanation is that we had insufficient sample sizes to fully characterize demographic differences. In the case where we modeled blubber cortisol with only the bycaught dolphin data represented (this was to remove fatality type as a factor), we had only six or fewer individuals to represent those states; resting (n = 5), pregnant (n = 6), lactating (n = 6). Analyzing dart biopsy samples taken directly from live free-ranging animals could help answer these questions, given that cortisol levels from these samples will not have any inherent signal from conditions leading to fatality; however, again, there is evidence that dart biopsying may change the composition of the blubber tissue. Any in-kind changes to cortisol levels would have to be assessed before studies with dart biopsies could be compared to those from dead carcass material.

This research marks a first step in validating blubber cortisol as a marker of stress (specifically activation of the HPA-axis) in cetaceans. The results of this study were supportive of this use. Whether blubber cortisol levels in bycaught and stranded animals were different due to activation of the HPA axis via 1) stress response (i.e., perception of threat-to-life) or 2) as a result of differences in nutritive condition before dying, the *a priori* expectation was that there should be large differences in blubber cortisol between these groups with stranded animals having more blubber cortisol than bycatch individuals. Though the distinguishing mechanism is still in question, we successfully demonstrated that we could measure these differences in the blubber. This suggests once baseline levels are effectively characterized that blubber cortisol may provide cetacean researchers with a tool to assess stress levels (activation of HPA axis) in free-ranging cetaceans. Moreover, because of the lag in signal before cortisol is integrated into the blubber, we may find it provides a way of assessing cortisol production with less influence of the act of sampling in the measured cortisol level compared with blood sampling.

## Supporting Information

S1 Data Non-normalized data for blubber cortisol and each covariate for every individual dolphin represented within this study. BLUBBER CORTISOL is the measured level of cortisol (ng) found per gram of blubber. For all two state categorical variables (e.g., PREGNANT) 1 = yes and 2 = no. For SEX, 1 = male and 2 = female. “nc” indicates data was not collected for that individual for that covariate. LATD = degrees latitude, LATM = minutes latitude, LONGD = degrees longitude, LONGM = minutes longitude. LENGTH = total length (cm) and ADRENAL WEIGHT = adrenal mass (g).
(CSV)
